# HSF1 overexpression enhances oncolytic effect of replicative adenovirus

**DOI:** 10.1186/1479-5876-8-44

**Published:** 2010-05-06

**Authors:** Cheng Wang, Zhehao Dai, Rong Fan, Youwen Deng, Guohua Lv, Guangxiu Lu

**Affiliations:** 1 Institute of Reproductive and Stem Cell Engineering, Central South University, Changsha 410008, China; 2 Institute of Spine Surgery, 2nd Xiangya Hospital of Central South University, Changsha 410007, China; 3 Department of Integrated Traditional Chinese and Western Medicine, Xiangya Hospital of Central South University, Changsha 410008, China; 4 Institute for Translational Medicine & Therapeutics, School of Medicine, University of Pennsylvania, Philadelphia, PA19104, USA

## Abstract

**Background:**

E1B55kD deleted oncolytic adenovirus was designed to achieve cancer-specific cytotoxicity, but showed limitations in clinical study. To find a method to increase its efficacy, we investigated the correlation between oncolytic effect of such oncolytic adenovirus Adel55 and intracellular heat shock transcription factor 1 (HSF1) activity.

**Methods:**

In the present study, human breast cancer cell line Bcap37 was stably transfected with constitutively active HSF1 (cHSF1) or HSF1 specific siRNA (HSF1i) to establish increased or decreased HSF1 expression levels. Cytotoxicity of Adel55 was analyzed in these cell lines *in vitro *and *in vivo*. Furthermore, Adel55 incorporated with cHSF1 (Adel55-cHSF1) was used to treat various tumor xenografts.

**Results:**

Adel55 could achieve more efficient oncolysis in cHSF1 transfected Bcap37 cells, both *in vitro *and *in vivo*. However, inhibition of HSF1 expression by HSF1i could rescue Bcap37 cell line from oncolysis by Adel55. A time course study of viral replication established a correlation between higher replication of Adel55 and cytolysis or tumor growth inhibition. Then, we constructed Adel55-cHSF1 for tumor gene therapy and demonstrated that it is more potent than Adel55 itself in oncolysis and replication in both Bcap37 and SW620 xenografts.

**Conclusions:**

cHSF1 enhances the Adel55 cell-killing potential through increasing the viral replication and is a potential therapeutic implication to augment the potential of E1B55kD deleted oncolytic adenovirus by increasing its burst.

## Background

Oncolytic adenoviruses are a class of promising anticancer agents, which are designed to selectively replicate in tumor cells and lead to cancer-specific cytotoxicity. Among them, a mutant adenovirus known as ONYX-015 is an elegant example, which was engineered to delete the E1B55kD viral protein and could preferentially replicate in p53-dysfunctional tumor cells but not the normal tissue [1,2]. Now, it shows unambiguous evidence of antitumor activity in a broader range of tumors than initially anticipated [3-6]. Although these oncolytic adenoviruses are promising as anticancer agents, clinical experiences show that these agents alone failed to generate sustained clinical responses or to cause complete tumor regressions. Better results could be achieved by combining the oncolytic adenoviruses with some chemotherapeutic agents (5-FU, cisplatin) [7] or antitumor transgene. However, we reasoned that to completely eradicate tumor cells, the replication efficacy of the oncolytic adenoviruses should be enhanced. Viral replication is dependent on the host cell microenvironment and requires redirection of the host cellular biochemical machinery by viral gene products to the favorable way. Various heat shock proteins (HSP) have been shown to be necessary for efficient adenovirus replication. Expression of human HSP70 has been shown to be stimulated during Ad5 infection [8,9], and HSP70 is expressed at high levels in Ad5-transformed human embryonic kidney cells (cell line 293) [9-11]. In recent studies, it has been demonstrated that the avian adenovirus CELO requires the induction of HSP40 and HSP70 for production of viral proteins and virions [12]. Previous studies showed that heat shock and heat shock protein 70 expression could enhance the oncolytic effect of replicative adenovirus in tumor cells [13]. Based on these evidences, we hypothesized that cells with higher HSPs expression might have more favorable environment for the replication of virus. To test our hypothesis, we explored the replication E1B55kD-deleted oncolytic adenovirus Adel55 in various tumor cells with different levels of HSPs transcription level. Our results showed the Adel55 oncolysis correlates with the HSPs transcription level.

Since Adel55 alone could not achieve efficient oncolysis in tumor cells, we designed a cell line with HSPs overexpression to further test the correlation between Adel55 oncolysis and HSPs transcription level. The expression of HSPs is accomplished through mechanisms that involve both transcriptional activation and preferential translation of heat shock transcription factor 1 (HSF1) [14,15]. HSF1 is present in the cytoplasm in an inactive, monomeric form. However, under stressful conditions, trimerization as well as phosphorylation occurs and HSF1 migrates to the nucleus, where it binds to a nucleotide recognition motif (nGAAn) within promoter/enhancer regions of HSP genes [14,15]. To overexpress HSPs in tumor cells, we used a HSF1 mutant with a deletion within the trimerization domain which can constitutively bind and transactivate HSP gene promoter [16]. Our results showed that this constitutive HSF1 mutant (cHSF1) overexpression could dramatically increase the replication of Adel55 in tumor cells and enhance the antitumor efficacy of Adel55 *in vitro *and *in vivo*. This finding leads us to treat tumor with oncolytic adenovirus harboring cHSF1, Adel55-cHSF1, and got more efficient oncolysis than Adel55 alone.

## Materials and methods

### Plasmids

Constitutively active heat shock factor 1 (cHSF1) with a deletion between amino acid positions 202~316 of wild-type HSF1 were generated by two step PCR as described previously [17]. cHSF1 PCR fragment were digested with *EcoR *I and subcloned into retrovirus vector LXSN and produced LcHSF1SN.

To inhibit the HSF1 expression, a 19-nt siRNA targeting to HSF1 gene was screened. The antisense fragment 5'-TCT CAA GGA GCT GCT CCT G-3'corresponding to nucleotide 322-340 of HSF1 was used. There is no homology of this fragment to other human genes found by BLAST assay. To express this fragment in plasmid, the pre-mirRNA was generated by ligating the following oligos through PCR without template: L-sense (5'-CTG ACA AGC TTG CTA AGC ACT TCG TGG CCG TCG ATC GTT TAA AGG GAG GTA GTG ATC TAG-3') and L-antisense (5'-CAG CAT ACA GCC TTC AGC AAG CCT CCA GGA ATT CAC TGT CTA GAT CAC TAC CTC CCT T-3'), HSF1i-fwd (5'-TGC TGA AGG CTG TAT GCT GTC TCA AGG AGC TGC TCC TGG TTT TGG CCA CTG ACT GAC G-3') and HSF1i-rvs (5'-GTA ACA GGC CTT GTG TCC TGT CTC AAG GAG CTG CTC CTG GTC AGT CAG TGG CCA AAA C-3'), R-sense (5'-CAG GAC ACA AGG CCT GTT ACT AGC ACT CAC ATG GAA CAA ATG GCC CAG ATC-3') and R-antisense (5'-ACT AGA AGC TTT AGA TAT TCT AGA TGC GGC CAG ATC TGG GCC ATT TGT TCC-3'). The product was named as HSF1i. HSF1i was digested by *Hind *III and cloned into LXSN, and produced LHSFiSN.

To construct a luciferase plasmid (HSE-Luc), hsp70B promoter was amplified from hsp70 cds using synthesized primers: 5'-GGA AGA TCT GAG AGT TCT GAG CAG G-3' containing a *Bgl *II restriction site and 5'-CCC AAG CTT TCC GGA CCC GTT GCC-3' containing a *Hind *III restriction site. The PCR fragment was subcloned into *Bgl *II-*Hind *III site of pGL3-enhancer vector (Promega).

### Virus construction

The E1B55kD gene deleted oncolytic adenovirus vector pAdel55 was established by nested PCR using pXC1 (Microbix Biosystems, Ontario, Canada) as the template. The viral region comprising nucleotides 1318-2038 was amplified using a primer set of 5 GCC GAC ATC ACC TGT GTC TAG AGA ATG -3' (L1) and 5'- TCA GAT GGG TTT CTT CAC TCC ATT TAT CCT-3' (R1). The region containing nucleotides 2005-2266 was amplified with another primer set of 5'-ATA AAG GAT AAA TGG AGT GAA GAA ACC CAT CTG AG-3' (L2) and 5'-GAA GAT CTA TAC AGT TAA GCC ACC TAT ACA ACA-3' (R2). Using the mixture of the two PCR products as template, a 955 bp fragment was then amplified using primers L1 and R2. This fragment was cut by *Xba *I and *Bgl *II and cloned into pXC1 to generate the plasmid pXC1-del55. SV40 polyA (160 bp) were obtained by PCR using pcDNA3 as temple and two primers: 5'-TGT GGA TCC TCT AGA GCT CGC TGA-3' and 5'-TCT AGA TCT CGA GCC CCA GCT GGT-3'. Then it was digested with *BamH *I and *Bgl *II and cloned into the *Bgl *II site of pXC1-del55 to generate the plasmid pAdel55. The correct construction of this vector was confirmed by DNA sequencing.

To generate pAdel55-EGFP, pAdel55-cHSF1, or pAdel55-HSF1i, EGFP, cHSF1 or HSF1i gene was cloned into the polycloning site of shuttle vector pCA13 first. Then the whole gene expression cassette was cut from pCA13-EGFP, pCA13-cHSF1 or pCA13-HSF1i by *Bgl *II and subcloned into the corresponding site of pAdel55.

Adenovirus was generated by standard homologous recombination techniques using the plasmid pAdel55, pAdel55-EGFP, pAdel55-cHSF1, or pAdel55-HSF1i and the adenovirus packaging plasmid pBHGE3 (adenovirus packaging plasmid, Microbix Biosystems, Ontario, Canada) in HEK293 cells. Recombinant adenovirus was isolated from a single plaque and expanded in HEK293 cells. A standard replication-deficient adenovirus Ad (E1A-) was generated using pCA13 and pBHGE3 for recombination. Viruses were plaque purified, propagated on HEK293 cells and purified by CsCl gradient according to standard techniques. Particle titers of all adenoviruses were determined by absorbance measurements at 260 nm, and functional PFU titers were determined by plaque assay on HEK293 cells.

### Cell lines and culture

Human hepatocarcinoma cell lines Hep3B, human cervical cancer cell line HeLa, human breast cancer cell line Bcap37, human colorectal cancer cell lines SW620, human normal amnion cells WISH, and human fetal lung fibroblasts HFL-1 were purchased from ATCC (American Tissue Culture Collection, Rockville, MD, USA). All cell lines were cultured in Dulbecco's modified Eagle's medium (DMEM; Gibco BRL) supplemented with 10% heat-inactivated fetal calf serum. The stable cell lines used in the experiment were established as follows. LXSN, LcHSF1SN or LHSF1iSN was transfected into Bcap37 cells by Lipofectamine (Life Technologies). After 24 h, cells were incubated in the selective media containing 500 μg/ml G418. The selection was continued for 14 days after which single cell colonies were picked and test using PCR. The selected stable cell lines, designated Bcap37/LXSN, Bcap37/cHSF1, and Bcap37/HSF1i were used for the experiments.

### Cytopathic effect assay

Cells were seeded at a density of 10^4 ^cells/well in a flat-bottomed 96-well. 24 h later, cells were treated with adenovirus as indicated. The 3-(4,5-dimethylthiazol-2-yl)-2,5-diphenyl tetrazolium bromide (MTT) assay was used to measure the viability of cells. Briefly, 20 ml MTT (5 mg/ml in PBS) was added to each well. After 4 h at 37°C, MTT was lightly removed and 100 ml of lysis buffer (10% SDS, 50% dimethyl formamide) was added. The plates were incubated for 7 h at 37°C before analysis on an ELISA reader at 595 nm.

### Luciferase assay

Cells were transfected with pGL3-Basic or pHSE-luc and pCMV-lacZ (Invitrogen) using Lipofectamine reagent (Life Technologies) according to the manufacturer's instructions. Luciferase assays were performed 48 h later using a Luciferase Assay System Freezer Pack Kit (Promega) and a luminometer. Internal normalization of the transfection efficacy was performed using a Luminescent Detection Kit (BD Biosciences) to detect β-galactosidase.

### Western blot

Cells were lysed in sodium dodecyl sulfatepolyacrylamide gel electrophoresis (SDS-PAGE) sample buffer (62.5 mM Tris-HCl, pH 6.8, 2% SDS, 10% glycerol, 1.55% DTT). Twenty micrograms of total protein was separated by 10% SDS-PAGE and transferred to nitrocellulose membranes (Amersham). Nonspecific binding on the nitrocellulose filter paper was minimized with a blocking buffer containing 5% nonfat dry milk in 13 TTBS (25 mM Tris-HCl, pH 7.5, 0.15 M NaCl, 0.05% Tween 20, 0.001% Thimerosal). The treated membrane was then incubated, first with the primary antibody mouse anti-human HSF1, Hsp90, HSP70, or HSP27 monoclonal antibody, or goat anti-human Actin polyclonal antibody serving as loading control and then with the secondary antibody horseradish peroxidase (HRP)-conjugated goat anti-mouse IgG or HRP-conjugated donkey anti-goat IgG. Both primary and secondary antibodies were purchased from Santa Cruz. The membrane was reacted with a chemiluminescent substrate (Pierce) according to the manufacturer's instruction and image was obtained by exposing an X-ray film.

### Viral replication assay

Cells or tumor tissues infected with adenovirus were collected and homogenized into single cell suspension in PBS at indicated time points. The adenoviruses were further released by 3 cycles of freeze-thaw. The homogenates were centrifuged at 12,000 g for 2 min and the supernatants were collected. The viral titer was determined by the standard plaque assay on HEK293 cells.

### In vivo tumor studies

All animal experiments were carried out in accordance with the National Institute of Health Guide for the Care and Use of Laboratory Animals. Five-week-old female BALB/c nu/nu mice were injected subcutaneously and bilaterally with 10^6 ^indicated cells suspended in 200 μl of serum free DMEM. Tumor growth was measured with calipers. Once the tumors reached about 3-4 mm in diameter, 10^8 ^PFU of Adel55 or Adel55-cHSF1 was suspended in 100 μl PBS and administered intratumorally. The perpendicular tumor diameter was measured every 5 days, and tumor volume (V) was calculated by the formula for a rotational ellipsoid: V (mm^3^) = length × width^2^/2.

### Immunohistochemistry assay

Paraffin tumor sections (8-10 μm) were treated with xylene, rehydrated in graded ethanol, and transferred to PBS. To detect the expression of HSF1 or Ad hexon, sections were incubated with anti-HSF1 antibody or anti-hexon antibody (Chemicon), followed by peroxidase-conjugated anti-mouse or anti-goat IgG (Santa Cruz Biotechnology). The staining signal was amplified by peroxidase-conjugated avidin-biotin complex (Vector Laboratories, Burlingame, CA, USA) and developed with DAB solution. Hematoxylin was used as counterstain. The stained sections were examined in a Zeiss photomicroscope (Carl Zeiss, Inc., Thornwood, NY, USA) equipped with a three-chip charge-coupled device color camera (Model DXC-960 MD; Sony Corp., Tokyo, Japan).

### Detection and quantification of apoptosis

Apoptotic cells in tumor specimens were detected using an in situ cell apoptosis detection kit (Promega) according to the manufacturer's instruction. Apoptotic cells were counted under a light microscope (400× magnification) in five randomly chosen fields, and the apoptosis index was calculated as a percentage of all cancer cells in these fields.

### Statistical analysis

Data are expressed as means ± SD values. Student's t test was applied to study the relationship between the different variables. Statistical significance was taken at *P *< 0.05.

## Results

### The oncolytic effect of Adel55 correlates with the intracellular HSF1 activity

**Figure 1:**
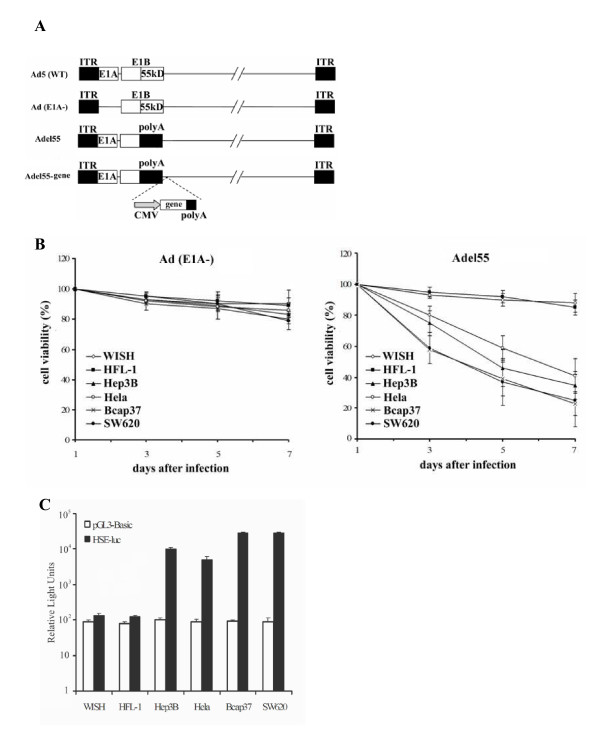
**The oncolytic effect of Adel55 correlates with the intracellular HSF1 activity**. (A) Schematic structure of Ad (E1A-), Adel55 and Adel55-gene. In Ad (E1A-), the E1A gene was deleted. In Adel55, the E1B55kDa gene was replaced by SV40 polyA. In Adel55-gene, the expression box of the foreign gene (EGFP, cHSF1, or HSF1i) was inserted into the *Bgl *II site after SV40 polyA. Wild type Ad5 structure was also depict. (B) The cytotoxicity of Ad (E1A-) and Adel55. Cells were infected with Ad (E1A-) or Adel55 at MOI of 10. Cell viability assay were performed by MTT assay at different time points. The cell survival rate of mock-infected cells was defined as 100%. The data represent the mean ± SD of three determinations. (C) Assessment of HSE activity in tumor or normal cell lines. Cells were transfected with luciferase reporter plasmid, pHSE-luc. Luciferase assays were performed 48 h later. The values (mean ± SD for four assays) are represented as relative light units/mg of protein. The cells transfected by pGL3-Basic without promoter were used as a negative control.

The oncolytic adenovirus we used for our study is Adel55, which was made by deleting the E1B55kD gene of the Ad5 and replacing it with polyA site. E1A-deleted replication defective adenovirus Ad (E1A-) was used in parallel as control (Figure 1A). To quantify the tumor cytopathic effect of Adel55, a set of tumor cell lines (Hep3B, Hela, Bcap37, SW620) and normal cell lines (WISH, HFL-1) were infected with Ad (E1A-) or Adel55 at MOI of 10, and cell viability was tested. As shown in Figure 1B, Ad (E1A-) caused no significant cell death to both tumor cell lines and normal cell lines. However, Adel55 had selectively cytotoxicity to all tumor cell lines tested, but not to normal cells. The cell viability rate of Bcap37 and SW620 cells infected with Adel55 was similar, which was around 10% less than that of Hep3B cells and 20% less than that of Hela cells (*P *< 0.05).

It has been reported that HSPs could enhance the replication of oncolytic adenovirus [12]. We hypothesized that the different cytotoxicity of Adel55 to different tumor cell lines might correlate to their different HSPs transcription level by HSF1. HSE is the promoter of the hsp70B gene, and HSF1 can bind to HSE and activate the transcription. Then, the HSE activity could reflect the HSF1 level in the cells. As shown in Figure 1C, Bcap37 and SW620 cell lines had the highest HSE activity, which is around 6-fold higher than Hela cells and 3-fold higher than Hep3B cells (*P *< 0.05). The HSE activity was corresponding to the cytotoxicity of Adel55. The higher is the HSE activity, the more cytotoxicity of Adel55 is to the cells. It suggested that the intracellular HSF1/HSE transcription level could affect the oncolytic effect of Adel55.

### HSF1 overexpression enhances the cytopathic effect and replication of Adel55 *in vitro*

**Figure 2:**
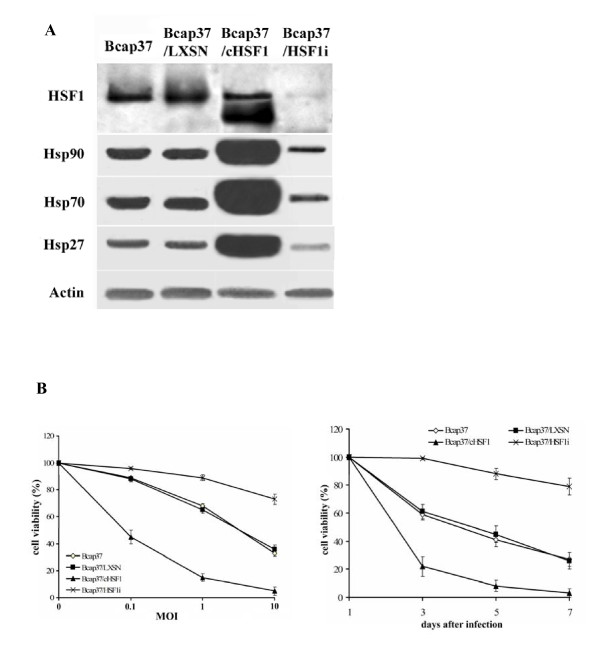
**HSF1 overexpression increases the oncolytic effect of Adel55 in Bcap37 cells**. (A) Western blot shows the levels of HSF1, Hsp90, Hsp70, Hsp27 in Bcap37 and the stable cell lines Bcap37/LXSN, Bcap37/cHSF1, Bcap37/HSF1i. Actin expression was used as a loading control. (B) The cytotoxicity of Adel55 on the indicated cell lines. Cells were infected with Adel55 at MOI of 0.1, 1, or 10. 7 days later, cell viability assay was performed. Meanwhile, another set of cells were infected with Adel55 at MOI of 10. 1, 3, 5 or 7 days later, cell viability assay was performed. The data represent the mean ± SD of four repeats.

To further confirm the correlation between HSF1 activity and the oncolytic effect of Adel55, a stable cell line, Bcap37/cHSF1, which overexpresses constitutively active HSF1 (cHSF1), and Bcap37/HSF1i, which inhibits the HSF1 expression by overexpressing the RNAi of HSF1, were used. Bcap37/LXSN which only contains the retroviral vector acted as control. Western blot was used to quantitate the HSF1-mediated Hsps transcription. The results showed HSF1i greatly reduced both HSF1 and HSPs expression. There's not much difference between Bcap37 and Bcap37/LXSN cells. Bcap37/cHSF1 cells have a higher HSF1 activity and strongly enhanced Hsps expression (Figure 2A).

To compare the cytotoxic effect of Adel55 to these stable cell lines, different MOI of Adel55 was used to infect these cells. As shown in Figure 2B, Adel55 could cause more significant cytopathic effect in Bcap37/cHSF1 than in Bcap37 and Bcap37/LXSN cell line at various MOIs. HSF1i expression in Bcap37/HSF1i strongly inhibited the cytopathic effect of Adel55. Similarly, when infect the cells with Adel55 at a MOI of 10 for various days, Bcap37/cHSF1 still shows the strongest cytopathic effect by Adel55. It suggests that the cHSF1 overexpression in Bcap37/cHSF1 cell line could enhance the cytopathic effect of Adel55, and inhibition of HSF1 by RNAi could rescue cells from the cytotoxicity by Adel55.

**Figure 3:**
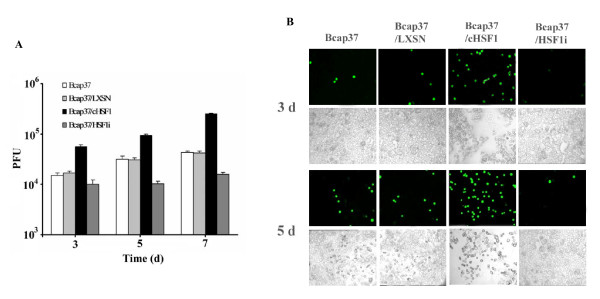
**HSF1 overexpression increases the replication of Adel55 in Bcap37 cells**. (A) Replication of Adel55 in the indicated cell lines. 10^5 ^cells were plated into six-well plates. 24 h later, cells were infected with 10^4 ^PFU of Adel55. After incubation for 3, 5, or 7 days, whole medium and cells extracts were prepared and titrated for virus production by standard plaque assay on 293 cells. The data represent the mean ± SD of four repeats. (B) The replication of Adel55-EGFP. Indicated cell lines were infected with Adel55-EGFP at MOI of 0.1. 3 or 5 days later, EGFP expression was detected under fluorescence microscopy (top panel). The corresponding cell death could be observed under bright field microscopy (bottom panel).

To investigate the mechanism of cHSF1-enhanced Adel55 oncolysis, we evaluated the patterns of viral replication. To this end, virus replication assay was performed. Adel55 at the MOI of 0.1 was used to infect cells and viral titer was tested on HEK293 cells at different time points after infection. As shown in Figure 3A, the replication of Adel55 in Bcap37/cHSF1 cells was higher than that in Bcap37 and Bcap37/LXSN cells. The same initial titer of Adel55 could produce around 10 folds of virus in Bcap37/cHSF1 cells than in Bcap37 and Bcap37/LXSN cells at day 7 after infection. Adel55 failed to replicate efficiently in Bcap37/HSF1i cells. This augmentation of viral replication in Bcap37/cHSF1 cells is in accordance with the increased oncolytic effect of Adel55. It indicates that cHSF1 overexpression could actually increase the replication of Adel55 and then leads to higher oncolysis.

To compare the replication and cytopathic effect of Adel55 in different cell line more directly, we used Adel55-EGFP to infect cells at a MOI of 0.1. As shown in Figure 3B, 3 or 5 days after Adel55-EGFP infection, Bcap37/cHSF1 cells have the highest EGFP expression level. Since EGFP expression is delivered by Adel55, its expression level could reflect the replication of Adel55-EGFP. The enhanced expression of EGFP in Bcap37/cHSF1 cells further confirmed that cHSF1 could increase the replication of Adel55-EGFP. The parallel observation under bright field microscope revealed the more efficient cytopathic effect of Adel55 to Bcap37/cHSF1 than to Bcap37 and Bcap37/LXSN. However, in Bcap37/HSF1i cell line, EGFP expression and cytopathic effect of Adel55 both got greatly inhibited. This further demonstrated that the cytopathic effect of Adel55-EGFP could be enhanced by cHSF1 overexpression.

### HSF1 overexpression enhances the antitumoral efficacy and replication of Adel55 *in vivo*

**Figure 4:**
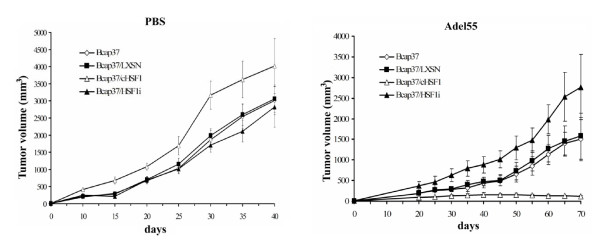
**HSF1 overexpression enhances the antitumoral efficacy of Adel55 in nude mice tumor model**. Five-week-old female BALB/c nu/nu mice were injected subcutaneously and bilaterally with 10^6 ^Bcap37, Bcap37/LXSN, Bcap37/cHSF1 or Bcap37/HSF1i cells suspended in 200 μl of serum free DMEM. When tumor volumes reached about 30 mm^3^, mice were intratumorally injected with PBS or 5×10^8 ^PFU of Adel55 once a day for 2 consecutive days. The tumor volume was measured at 5-day intervals and is presented as means ± SD of eight mice.

To investigate whether cHSF1 could also enhance the oncolytic efficacy of Adel55 *in vivo*, xenograft models generated from different cell lines were treated by Adel55. As shown in Figure 4, after PBS injection, all the tumors grew rapidly. Bcap37/cHSF1 xenograft showed higher growth rate than the other three. It might be due to the overexpression of HSPs being able to favor tumor growth. However, after 20 days of treatment, Adel55 clearly showed great inhibition of all tumor growth compared with the PBS treated group. Especially for Bcap37/cHSF1, the tumor volume was maintained below 150 mm^3 ^and slowly decreased during the process. Bcap37 and Bcap37/LXSN xenografts got relatively rapid growth rate after Adel55 treatment, while Bcap37/HSF1i grew even faster, with double the tumor volume of Bcap37 at the end of the experimental period. Then, cHSF1 overexpression could also enhance the oncolytic effect of Adel55 *in vivo*.

**Figure 5:**
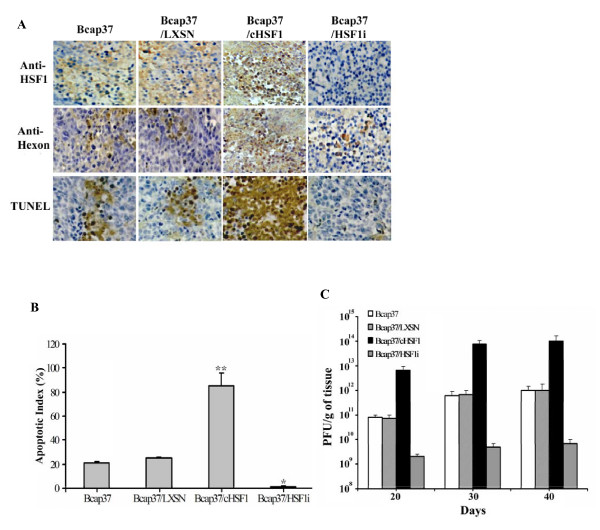
**HSF1 overexpression enhances the oncolytic effect of Adel55 in nude mice tumor model by increasing Adel55 spreading and tumor apoptosis**. (A) Detection of HSF1 expression, Adel55 spreading or apoptotic rates in tumors. 30 days after injection, cHSF1 and Adel55 hexon expression in each treated group was detected by immunostaining with anti-HSF1 or anti-hexon antibody, respectively. Apoptotic rate in each group was detected by an in situ cell apoptosis detection kit. (B) The apoptotic index of the mean values of sections from three mice in each group and are presented as means ± SD (n = 3; *P < 0.05, Bcap37/HSF1i vs Bcap37 or Bcap37/LXSN; **P < 0.01, Bcap37/cHSF1 vs other groups). (C) The replication of Adel55 *in vivo*. The Adel55 virus progeny recovered from the xenografts was titrated for virus production by standard plaque assay on 293 cells. The titer was adjusted to the weight of each tumor and expressed as PFU/g tissue. Each column represents the mean titer ± SD (n = 3).

To analyze the distribution of Adel55 in the tumor tissue, we did the tumor section histological examination. As shown in Figure 5A, 30 days after Adel55 injection, Bcap37/cHSF1 has much higher HSF1 expression than Bcap37 and Bcap37/LXSN. There is no obvious HSF1 detected in Bcap37/HSF1i tumor. It is consistent with the western blot result *in vitro*. Hexon of the adenovirus capsid was immune-stained to detect the distribution of Adel55 in the tumor tissues. In Bcap37/cHSF1 group, Adel55 got extensive expansion throughout the whole section. While Bcap37 and Bcap37/LXSN only got partial sections stained positively. HSF1i expression even suppressed the Adel55 expansion. This suggests that Adel55 could replicate efficiently in HSF1 overexpression tumor tissue, which is consistent with the *in vitro *result. According to previous studies, oncolytic adenovirus could lead to the apoptosis of tumor cells, so we also test the apoptotic ratio in different groups after Adel55 treatment. The TUNEL results showed that compared to the low apoptotic ratio in Bcap37 and Bcap37/LXSN tumor cells, Adel55 could induce efficient apoptosis of Bcap37/cHSF1 tumor, while apoptosis was not obvious in Bcap37/HSF1i group. The quantization of apoptosis ratio showed that the apoptotic index in Bcap37/cHSF1 group is four times higher than Bcap37 and Bcap37/LXSN groups. The apoptotic index in Bcap37/HSF1i group is very low (Figure 5B). These results further demonstrated that Adel55 could replicate and expand efficiently in tumor cells with higher HSF1 activity, which could lead to effective oncolysis.

Consistent with *in vitro *data, the quantization of Adel55 replication *in vivo *also showed cHSF1 enhanced the oncolysis of Adel55 through augmenting its replication (Figure 5C). The viral titers from Bcap37/cHSF1 xenografts were around 100 folds higher than those from Bcap37 and Bcap37/LXSN xenografts. The titer of Adel55 kept increasing over time. Inhibition of HSF1 expression in Bcap37/HSF1i could also inhibit the Adel55 replication.

### Adel55-cHSF1 can achieve higher antitumoral efficacy

**Figure 6:**
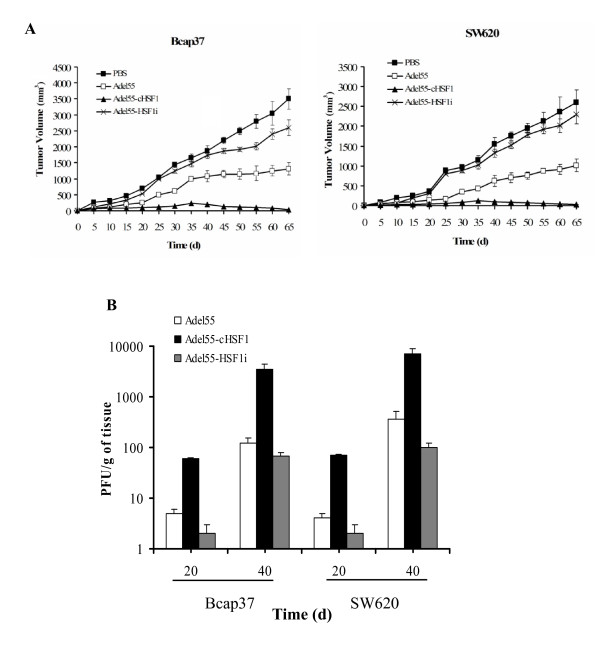
**The antitumor efficacy of Adel55-cHSF1 on Bcap37 and SW620 xenograft tumors in nude mice**. (A) The antitumoral efficacy of Adel55-cHSF1 in nude mice bearing Bcap37 or SW620 xenograft tumors. When tumor volumes reached about 30 mm^3^, mice were intratumorally injected with PBS or 5×10^8 ^PFU of Adel55, Adel55-cHSF1 or Adel55-HSF1i once a day for 2 consecutive days. The tumor volume was measured at 5-day intervals and is presented as means ± SD of eight mice. (B) Titration of virus progeny recovered from tumor xenografts of 20 or 40 days. Extracts were prepared and titrated as described in Materials and Methods. The titer was adjusted to the weight of each tumor and expressed as PFU/g of tissue. Each column represents the mean titer ± SD (n = 3).

On the basis of the studies described above, we applied the favorable effect of cHSF1 on oncolytic adenovirus for tumor gene therapy *in vivo*. To this end, cHSF1 gene was inserted into the oncolytic adenovirus Adel55 and administered intratumorally to Bcap37 or SW620 xenograft. As shown in Figure 6A, compared to Adel55, Adel55-cHSF1 showed more effective antitumoral efficacy in both Bcap37 and SW620 tumors, and tumors stopped growing and disappeared around day 65. Tumors treated with PBS or Adel55-HSF1i grew much faster. It indicated that Adel55-cHSF1 could more efficiently mediate oncolysis of tumor cells of various origins.

The virus replication assay at day 20 and day 40 showed that the titer of Adel55-cHSF1 in tumor tissue is 50~100 times higher than that of Adel55 or Adel55-HSF1i (Figure 6B). This further demonstrated that Adel55-cHSF1 could lead to overexpression of HSF1 in tumor tissues and then enhance the replication of Adel55 to kill the tumor cells more efficiently. Both Adel55 and Adel55-cHSF1 showed around 20% more efficient oncolytic effect in SW260 tumor than in Bcap37 tumor, which was probably due to other intracellular environment critical for tumor progression.

## Discussion

Cancer gene therapy is currently limited by the inadequacy of vectors to completely eliminate the malignant clone. Oncolytic adenovirus has been shown to selectively lyse tumor cells and replicate efficiently throughout the tumor mass. However, clinical results of using oncolytic adenovirus as a therapeutic agent clearly indicated improvement is needed to achieve significant antitumoral activity. Previous studies indicated HSPs may play potential role in the replication of oncolytic adenovirus [18-21]. ONYX-015 is an E1B55kD deleted adenovirus that has promising clinical activity for cancer therapy. However, many tumor cells fail to support ONYX-015 oncolytic replication. E1B55kD functions include p53 degradation, RNA export, and host protein shutoff. Previous study demonstrated that resistant tumor cell lines fail to provide the RNA export functions of E1B55kD necessary for ONYX-015 replication; viral 100 K mRNA export is necessary for host protein shutoff. However, heat shock rescues late viral RNA export and renders refractory tumor cells permissive to ONYX-015. It suggests that heat shock and late adenoviral RNAs may converge upon a common mechanism for their export and the concomitant induction of a heat shock response could significantly improve ONYX-015 cancer therapy [22].

Therefore, we hypothesized that heat shock transcription level in cells might affect viral replication. To test our hypothesis, we employed four tumor cell lines of different origins and tested the effect of different HSF1 activity on the oncolytic effect of an E1B55kD gene deleted oncolytic adenovirus Adel55 constructed by our lab. We found Adel55 could replicate more efficiently and mediate more effective cytotoxicity in tumor cells with higher level of HSF1 activity.

To further confirm the correlation between HSF1 level and replication of Adel55, a constitutively active HSF1 gene, cHSF1, was evenly overexpressed in Bcap37 cells by establishing a stable cell line, Bcap37/cHSF1. Increased oncolytic effect and replication of Adel55 was detected on Bcap37/cHSF1 *in vitro *and *in vivo*, and its oncolytic effect was directly related to its replication. It confirmed the correlation between HSF1 activity and the viral replication of Adel55. Interestingly, Adel55-cHSF1 could achieve a much higher oncolytic efficacy from various origins in a mouse model. These results confirmed the importance of intracellular HSF1 level for the replication of oncolytic adenovirus.

The possible mechanism of why cHSF1 overexpression can increase the oncolytic effect of Adel55 may involve the natural role of cHSF1 in cells. We reasoned that cHSF1 could induce the overexpression of HSPs. The replication of adenovirus DNA may be dependent on HSP [12]. Importantly, in the late stage of adenovirus infection, the translation of the host cell proteins is generally stopped, while the HSP mRNA transcription is maintained. This indicated that the synchronized expression of HSP in adenovirus infected cells may confer a selective advantage for the viral life cycle [21]. On the other hand, cHSF1 overexpression may change the expression level of some other genes which are responsible for adenovirus life cycle in cells, such as Coxsackie virus and adenovirus receptor (CAR), which sequentially affects the infection and replication of adenovirus.

In this study, we applied the heat shock transcription mechanism and induced a favorable inner environment for adenovirus DNA replication through overexpressing cHSF1. Our results demonstrated that cHSF1 overexpression selectively increase the oncolytic effect and replication of oncolytic adenovirus Adel55 *in vitro *and *in vivo*. On the other hand, when applied to cancer gene therapy in immune-competitive host, cHSF1 overexpression could also induce the tumor-specific immune response. We have tested the therapeutic effect of Adel55-cHSF1 in normal mice bearing rodent tumors and enhanced tumor specific immune response was detected (Wang C, unpublished data).

## Conclusions

In conclusion, intratumoral HSF1 level is positively correlated with the oncolytic effect of E1B55kD deleted adenovirus. The advantage of Adel55-cHSF1 for tumor gene therapy includes the enhanced oncolytic adenovirus replication and induction of tumor-specific immune response. It paved a road to the combined application of heat shock pathway and oncolytic adenovirus for tumor treatment in the future.

## Competing interests

The authors declare that they have no competing interests.

## Authors' contributions

CW carried out all the *in vitro *experiments, and was primarily responsible for drafting the manuscript. ZD and RF carried out the xenografts model establishment and collaborated in immunohistochemistry assay and apoptotic detection of tumor tissues. YD and GLv collaborated in some xenografts model establishment. GLu participated in the design of the study and collaborated in the drafting the manuscript.

All authors have read and approved the final manuscript.
